# Breast cancer in pregnancy: an institutional experience

**DOI:** 10.3332/ecancer.2015.551

**Published:** 2015-07-08

**Authors:** Abraham Hernández Blanquisett, Carmen Herrero Vicent, Joaquín Gavilá Gregori, Ángel Guerrero Zotano, Vicente Guillem Porta, Amparo Ruiz Simón

**Affiliations:** Department of Medical Oncology, Fundación Instituto Valenciano de Oncología, Valencia 46009, Spain

**Keywords:** pregnancy, breast cancer, postpartum, delivery

## Abstract

**Background:**

Breast cancer is one of the most common cancers diagnosed during pregnancy. Pregnancy-associated breast cancer (PABC) is defined as breast cancer diagnosed during pregnancy or within 12 months of delivery. Nowadays PABC can be safely diagnosed, staged, and treated during pregnancy with good outcomes for both the mother and the fetus. Recent studies suggest that prognosis of women diagnosed during postpartum seems to be worse. In order to gain a better understanding of the PABC, we reviewed our centre’s experience.

**Patients and methods:**

We assessed the clinicopathological parameters, evolution, and outcome of patients treated in the Fundación Instituto Valenciano de Oncología of Valencia, Spain, from October 1990 to October 2013, and compared the results of patients diagnosed during pregnancy (group ‘A’) and patients diagnosed within one year of delivery (group ‘B’).

Of 12,000 cases of breast cancer registered in our database, 35 cases of PABC were identified. We included 11 patients in group ‘A’ and 24 in group ‘B’.

**Results:**

In our group the median age was 35 years (range 29–42), of which ten (28%) patients had family history (first grade) of breast cancer, four patients were BRCA 1 mutation carriers. Axillary node compromise was found in 19 patients (53.5%), 24 patients were stage II or III at diagnosis (68.5%), 22 (62.8%) were ER positive, and nine (25.7%) were HER-2 positive.

In group A (n = 11), five patients diagnosed before 18th week decided that a therapeutic abortion be performed before treatment, two patients were treated during pregnancy, one with chemotherapy without treatment associated complications during delivery. Four women diagnosed after 28th week decided to delay the treatment until delivery.

After a follow up of 172 months, the relapse free survival (RFS) was 69% at five years and 45% at ten years. Overall survival (OS) at five years was 90.8% and 74.2% at ten years for all patients. For group ‘A’ OS was higher with 90% at five years versus 80% in group ‘B’. The differences between the groups were not statistically significant p = 0.368.

**Conclusion:**

In our experience, there is a higher OS in patients diagnosed during pregnancy suggesting a better prognosis for this group of women but the difference between the groups is not statistically significant. Our study is limited because of our small sample.

## Introduction

Breast cancer is one of the most common cancers diagnosed during pregnancy along with melanoma and cervical cancer, with reported frequencies of one in 1000 to three in 10,000 pregnancies [1]. It is expected to become even more common, since these days women often delay childbearing to their 30s and 40s when breast cancer rates tend to increase [2]. However, the diagnosis of cancer during pregnancy is still an uncommon event.

PABC has been traditionally associated with a worse prognosis and OS [3, 4, 5]. Most of women with PABC present larger tumours and have higher incidence of lymph node metastasis at diagnosis [6]. The reason might be because of a delay in diagnosis. This could be explained by engorgement and physiologic hypertrophy of the pregnant or lactating breast that creates difficulties in interpreting clinical examinations and mammography (increased breast density).

Unfortunately, there is a knowledge gap for understanding whether pregnancy and other prognostic factors affect the survival of women with PABC.

The objective of this report is to analyse the clinicopathological characteristic and prognosis of patients diagnosed with PABC and compare the outcomes in women diagnosed during pregnancy with those diagnosed in the postpartum period, because the prognosis of this group seems to be more worse.

## Patients and method

In this descriptive study we assessed the clinicopathological parameters, evolution, treatment, and prognosis of patients with PABC and compared the results of patients diagnosed during pregnancy (group ‘A’) and patients diagnosed within one year of delivery (group ‘B’), treated in the Fundación Instituto Valenciano de Oncología (I.V.O) in Valencia, Spain.

We defined PABC as the breast cancer presented during pregnancy or up to one year after delivery. Of 12,000 cases of breast cancer registered in our database, we identified 35 cases of PABC. The histological grading was performed using the Scarff-Boom-Richardson (SBR) histological system. For the study of ER and PR a nuclear staining >1% of tumour cell was considered as positive. Patients were considered HER-2 positive if immunohistochemistry (IHC) was positive (+++) or HER-2 was amplified by FISH (Fluorescence *in situ* hybridisation). In our sample, we had three Patients with (++) for HER-2 IHC, and FISH was not performed to confirm their HER-2 status. We considered them as HER-2 negative for the analysis.

We recorded age at diagnosis, time from the beginning of the symptoms until diagnosis, tumour pathological findings, hormonal receptors, and HER-2 status, type of surgery, use of systemic therapy, and hormonal therapy. The characteristics of the patients are described in Table 1.

OS was defined as the time period from initial diagnostic biopsy until death from any cause. RFS was defined as time period from diagnosis to first relapse (locoregional or systemic).

We also compared the outcome between patients diagnosed during pregnancy with those diagnosed after delivery. Data were analysed with SPSS 22.0 software. Survival curves were calculated according to the Kaplan-Meier analysis and compared with the log-rank test. A ‘p’ value <0.05 was considered statistically different.

## Results

### Baseline characteristics

From October 1990 to October 2013, we found prevalence for PABC of 0.29%. The median age was 35 years (range 29–42). The median time between the first symptom and diagnosis was 5.2 months (range 0–15 months). Among them ten (28%) patients had first grade family history of breast cancer, mutational study for BRCA was performed in 12 patients, four were BRCA 1 mutation carriers, and five more patients had variants of uncertain significance (VUS) for BRCA 1 or BRCA 2.

Axillary node compromise was found in 19 patients (53.5%), 24 patients (68.5%) were diagnosed in stage II or III, and three patients in stage IV.

The most common histological type was invasive ductal carcinoma found in 25 patients (71.4%), 11 were classified as grade 2, and eight as grade 3 according to SBR histological grading scale.

The status of oestrogenic and progesterone receptor was known in 32 patients (91%), of which 22 were ER positive (62.8%). The HER-2 status was studied in 29 (82%) patients. We found nine HER-2 positive (25.7%). We also found five triple-negative tumours.

### Treatments

Surgery was performed in 87.7% of cases; total mastectomy (53.5%), partial mastectomy (34.2%). Among these 80% were treated with anthracycline and taxane-based chemotherapy. A total of 77% received local radiotherapy and 62.8% of patients received hormonal therapy, mostly with tamoxifen (86.3%).

In group A (n = 11), five patients diagnosed before 18th week decided that therapeutic abortion be performed before treatment, four patients diagnosed after 28th week decided to delay the treatment until delivery, and two patients were treated during pregnancy. One was treated with total mastectomy at 28th week without complications.

The other woman with a twin pregnancy was diagnosed in the 12th week of gestation; she decided to start treatment, total mastectomy was performed at 13th week of gestation without complications. Adjuvant treatment with doxorubicin and cyclophosphamide was started at 21th week of gestation with nausea and vomiting grade 1 as only side effect. After the third cycle she had grade 4 toxicity (neutropaenia and mucositis). We decided to stop adjuvant treatment at 24th week of gestation, the ultrasound revealed an appropriate weight for the gestational age at this moment. A planned cesarean delivery was performed at 32th week of gestation because of intrauterine growth restriction diagnosed in the ultrasound scan. The twins were born with low weight (1200 g and 1400 g) with an Apgar score of 7/10 and 10/10; one of them had to be taken to the intensive care unit for a few days for a respiratory infection. After two years of follow up, the girls had a normal weight for her age (75th percentile), at last follow-up (12 years) they had not shown any complication associated with chemotherapy treatment.

In group B (n = 24), 21 women were treated by surgery, 50% with total mastectomy, and 37.5% with partial surgery. A total of 20 received chemotherapy and 17 hormonal therapies.

### Efficacy results

The median follow-up was 172 months for the total group. At last follow-up 11 patients had relapsed (31.4%). The RFS was 69% at five years and 45% at ten years of follow-up, with a median of 32 months. The difference between the groups was not statistically significant.

**Figure 1.**
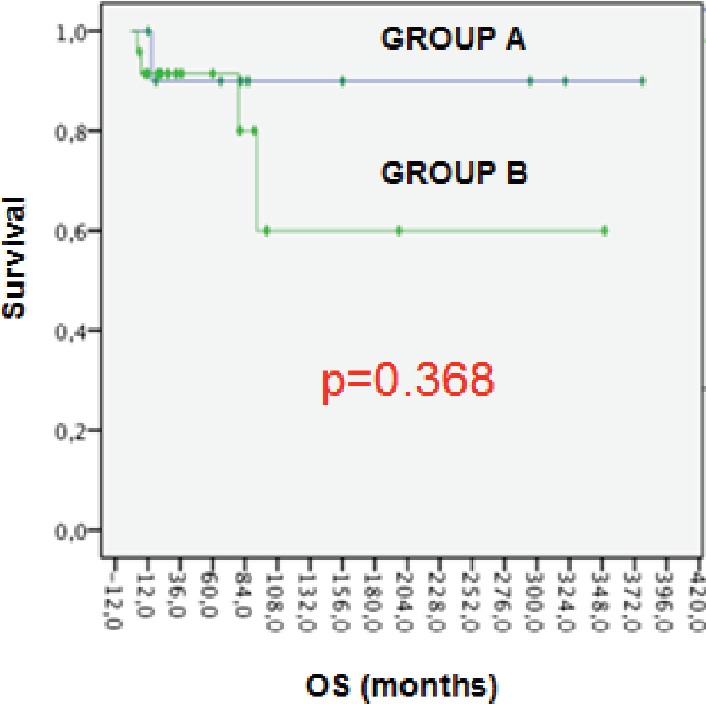
OS for groups.

Five patients (14.2%) had died and all of them because of breast cancer. OS at five years was 90.8% and 74.2% at ten years for all patients. For group ‘A’ OS was higher with 90% at five years versus 80% in group ‘B’, but the differences between the groups were not statistically significant (p = 0.368). (Figure 1).

**Figure 2.**
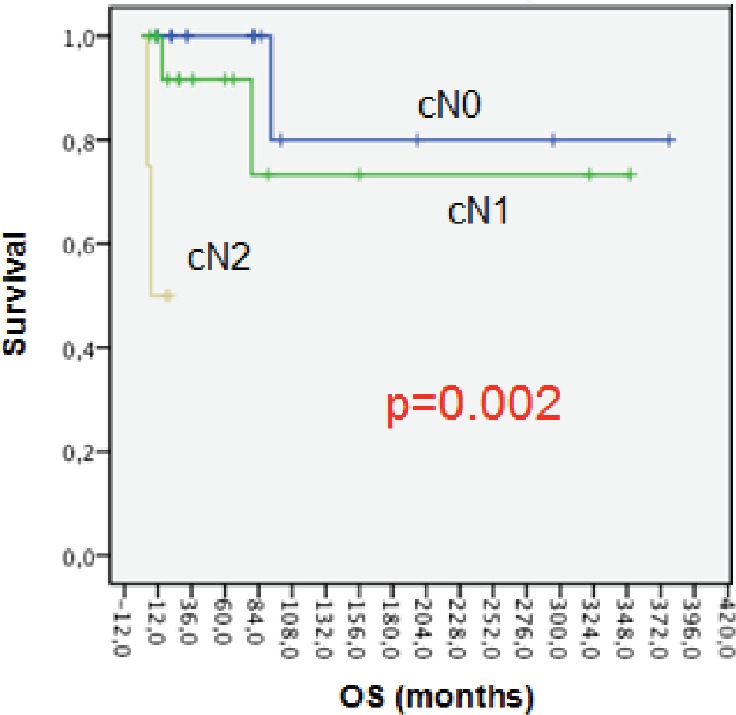
OS axillary node compromise.

Lower OS at five years was associated with patients with axillary node compromise at diagnosis (Figure 2). OS at five years was 100% for patients with cN0; 91% for cN1 patients, and 0% for cN2 with p = 0.002. The differences can be explained because we only included four patients in cN2.

## Discussion

Breast cancer occurring during pregnancy or within first year after delivery is considered to be PABC. PABC is relatively rare and its incidence is 0.2–3.8% [7]. In our study PABC constitutes 0.29% of total cases of breast cancer patients.

The frequency of PABC is increasing. In recent data published by Beadle *et al*, in young breast cancer patients, out of 668 patients analysed, 104 were PABC which constitute up to 15% of cases [8]. This study was made in a tertiary centre for referral of pregnant patients and that can explain their high frequency of cases. However, the delay in the age at first pregnancy could explain why the frequency on PABC has increased, mainly in developed countries.

The median age of diagnosis for our patients was 35 years (range: 29–42 years) similar age was reported by Sanchez *et al* [9]. In previous studies, PABC was associated with a genetic predisposition and a strong family history [10, 11]. We found that 28% of our patients had a positive family history and four patients were BRCA 1 mutation carriers, and five more patients had variants of uncertain significance (VUS) for BRCA 1 or BRCA 2.

The diagnosis of PABC may be difficult because the anatomic changes of the breast. Mostly of women who are diagnosed in locally advanced stages (68% in our database), with poorly differentiated tumours, are often ER negative [1, 3]. The delay in diagnosis is probably explained by the difficulties in the physical examination and the general assumption that a tumour in breast is benign and possibly related to pregnancy. In a recent study published by Gogia *et al* the median duration of symptoms and diagnosis was 11.5 months [12]. Our median duration between the first symptom and diagnosis was 5.2 months (range 0–15 months).

Numerous studies have found that PABC was associated with larger tumours and positive axillary nodes, from 56% to 83% of pregnant women at diagnosis versus 38% to 54% in non-pregnant population [13, 15]. In our study axillary node compromise was found in 53.5% of the cases. In contrast to earlier studies, in this report we found higher ER/PR expression (62.8%) and lower HER-2/neu overexpression (25.7%) [13, 16, 17].

Treatment of the disease during pregnancy with surgery and chemotherapy is effective and feasible. The use of chemotherapy after the first trimester has been widely reported based on schemes such as anthracyclines and taxanes [1, 4], which has been used in our patient.

The decision to terminate a pregnancy during breast cancer is a highly personal decision to be made by an informed woman. A woman may be advised to terminate a pregnancy if it is felt that her life expectancy may not be longer than gestation. More recently the frequency of the recommendation for termination has been decreasing, regarding the similar prognosis for the pregnant patient, and the safety of surgery and chemotherapy during pregnancy [1]. In addition, termination of the pregnancy has not been shown to improve survival. In our study, five patients diagnosed before 18th week decided that therapeutic abortion was performed before treatment. We treated two patients during pregnancy with good outcomes for the mothers and the newborns.

PABC has traditionally been associated with a worse prognosis and OS [3, 4, 5]. Azim *et al* reported in their metaanalysis of PABC that there was a poorer breast cancer outcome for women diagnosed in the postpartum period compared with those diagnosed with breast cancer during pregnancy [5]. Amant *et al* analysed the prognosis of 311 women with breast cancer, who were diagnosed and treated during pregnancy. Patients diagnosed with breast cancer postpartum were excluded from this analysis. Interestingly, no differences in mortality were found between this group and a cohort of the same age with breast cancer unrelated to pregnancy [18]. These findings suggest that pregnancy has no negative impact on breast cancer giving relevant information for counselling women who are diagnosed during pregnancy [19].

However, the same conclusion is not extended for patients with breast cancer diagnosed in the postpartum period. In this situation, the prognosis seems to be more worse [20, 21]. Many studies in the past have considered the two groups (breast cancer during pregnancy and postpartum breast cancer) as part of the same condition, and this could be the reason for the controversial results on prognosis [22].

In our study, we reported a RFS of 69% and 45% at five and ten years with a median of 32 months and we estimated an OS of 90% and 74% at five and ten years. Prognosis in patients with breast cancer diagnosed in the postpartum period seems to be more worse [20, 2]. We included both populations; breast cancer diagnosed during pregnancy (group ‘A’) and after 1st year of delivery (group ‘B’). Interestingly, for group ‘A’ OS was higher with 90% at five years versus 80% in group ‘B’, but the difference found between the groups was not statistically significant. The limitations of our study are mainly based on the retrospective nature of the data and the small sample size.

## Conclusions

Nowadays PABC can be safely diagnosed, staged, and treated during pregnancy with good outcomes. We have successfully treated two patients during pregnancy, and those patients did not present any complications associated with treatments during delivery and their children are growing up healthy.

In our experience, there is a higher OS in patients diagnosed during pregnancy suggesting a better prognosis for this group of women. The difference found between the groups was not statistically significant. Our study is limited by the small number of patients and we cannot make further conclusions.

We need more translational studies for a better understanding of PABC biology leading to a better classification of groups of poor prognosis.

We conclude that once a pregnant woman is diagnosed with breast cancer she should be referred to receive a multidisciplinary approach and personalised treatments looking for the best outcomes for both the mother and the fetus.

## Figures and Tables

**Table 1.** Patients’ characteristics.

Patients’ and disease characteristics	Group A (*n* = 11)	Group B (*n* = 24)	Total (*n* = 35)
**cT 2-3-4**	8	18	26
**cN 0**	6	10	16
**cN 1-2**	5	14	19
**Stage II–III**	7	17	24
**Stage IV**	0	3	3
**CDI**	8	17	25
**Grade 2**	3	8	11
**Grade 3**	3	5	8
**ER/RP positive**	5	17	22
**HER-2 positive**	1	8	9
**Partial mastectomy**	3	9	12
**Total mastectomy**	7	12	19
**Anthracycline and taxane**	8	20	28
**Relapse**	3	8	11
**Exitus**	1	4	5
